# Curcumin in Combination with Other Adjunct Therapies for Brain Tumor Treatment: Existing Knowledge and Blueprint for Future Research

**DOI:** 10.22088/IJMCM.BUMS.10.3.163

**Published:** 2022-01-10

**Authors:** Kavita Peter, Santosh Kumar Kar, Ragini Gothalwal, Puneet Gandhi

**Affiliations:** 1 *Department of Biotechnology, Barkatullah University, Bhopal, M.P, India.*; 2 *Nano Herb Research Lab, KIIT-TBI, Bhubaneshwar, Odisha, India.*; 3 *Department of Research, Bhopal Memorial Hospital and Research Centre, Bhopal, M.P, India.*

**Keywords:** Glioblastoma, polyphenol, combination therapy, resistance, sensitization, synergistic

## Abstract

Malignant brain tumors proliferate aggressively and have a debilitating outcome. Surgery followed by chemo-radiotherapy has been the standard procedure of care since 2005 but issues of therapeutic toxicity and relapse still remain unaddressed. Repurposing of drugs to develop novel combinations that can augment existing treatment regimens for brain tumors is the need of the hour. Herein, we discuss studies documenting the use of curcumin as an adjuvant to conventional and alternative therapies for brain tumors. Comprehensive analysis of data suggests that curcumin together with available therapies can generate a synergistic action achieved through multiple molecular targeting, which results in simultaneous inhibition of tumor growth, and reduced treatment-induced toxicity as well as resistance. The review also highlights approaches to increase bioavailability and bioaccumulation of drugs when co-delivered with curcumin using nano-cargos. Despite substantial preclinical work on radio-chemo sensitizing effects of curcumin, to date, there is only a single clinical report on brain tumors. Based on available lab evidence, it is proposed that antibody-conjugated nano-curcumin in combination with sub-toxic doses of conventional or repurposed therapeutics should be designed and tested in clinical studies. This will increase tumor targeting, the bioavailability of the drug combination, reduce therapy resistance, and tumor recurrence through modulation of aberrant signaling cascades; thus improving clinical outcomes in brain malignancies.

Malignant brain tumors have an unvaryingly bleak prognosis. According to the last brain and central nervous system (CNS) cancer statistics released by GLOBOCAN; 308,102 new cases and 251,329 deaths were reported worldwide in 2020 (1). Since 2005, Stupp care protocol has been the standard line of treatment for brain tumors, like glioblastoma (GB), the most aggressive grade IV glioma (2). Looking at the treatment outcomes in the last 15 years, in terms of survival and quality of life of patients, this therapy regimen needs improvement. The major hitches in brain tumor treatment are: lack of gross total surgical resection due to the tumor location, presence of the blood-brain barrier (BBB) that doesn’t allow the entry of majority of the chemotherapeutic agents into the brain, infiltrative nature of the tumor, toxicity, and resistance induced by the prolonged administration of chemo-radiotherapy. This clinical scenario necessitates the repurposing of established, non-toxic therapeutic agents as an adjunct to overcome treatment-induced resistance, reduce its side effects, and prevent a recurrence. 

The present century has witnessed a resurrection of interest in complementary medicine for the development of alternative agents in the treatment of neoplasms. Currently, few of the primary and adjuvant therapies being explored for brain cancers like resveratrol, curcummin (CUR), berberine, quercetin, genistein, etc (3-7) are derived from natural sources. These nutraceuticals are functional foods with anti-cancerous propensities due to their anti-oxidant and anti-inflammatory character. Among these CUR has stood out as a strong contender as for centuries, turmeric has been used in the Indian Ayurvedic system due to its potent anti-inflammatory, antioxidant and anti-infective properties. It is the principal curcuminoid (77%) derived from the rhizome of the perennial herb *Curcuma longa* (8). The rhizome is commonly known as turmeric, which is an integral spice of almost all Indian cuisines. For centuries, turmeric has been used in the Indian Ayurvedic system due to its potent anti-inflammatory, antioxidant and anti-infective properties. However, its anti-cancerous properties were deciphered in 1985 when Kuttan and colleagues, published their first positive preclinical report on its active constituent ‘CUR’ (9). This was further validated by their clinical study (10). Since then CUR has been reported for its anti-cancerous effects in various cancers including brain tumors (11, 12). CUR is now known to be toxic to the cancer cells, with minimal or no toxicity towards normal cells (13, 14). Its anti-cancerous properties are attributed to its pleiotropic biological effects viz. inhibition of proliferation, survival, invasion, angiogenesis, metastasis, epithelial to mesenchymal transition (EMT), and cancer stem cells (CSCs) differentiation. The dietary polyphenol has shown an exceptional safety profile in clinical trials; being nontoxic even at a dose of 8 g/day (15). All these factors put together endorse its use as an adjunct to conventional therapies. The US Food and Drug Administration (FDA), has approved the yellow spice as GRAS (generally recognized as safe). 

Treatment with single agents, whether synthetic or natural, has limitations since brain tumors present intra-tumor genomic and cellular heterogeneity (16). Therefore, the focus of current research is on repurposing established chemotherapeutic agents and the use of drug combinations for successful patient outcomes. A growing body of evidence has shown that CUR can potentiate treatment endpoints when used in combination with other therapeutic agents in brain tumors (17). A literature search was carried out wherein both *in vitro* and *in vivo* studies using CUR in combination therapy on brain tumors were included. Studies, in which CUR was encapsulated along with other chemotherapeutic agents within nanoparticles to increase bioavailability, were also incorporated. This review comprehensively summarizes the potential therapeutic approaches and molecular mechanisms where CUR is administered with standard (Figure 1) and alternative treatment modalities in brain malignancies. The rationale of the present study was to endorse the concept of CUR in combination therapy for brain cancers, based on the current WHO guidelines of molecular diagnosis to target multiple tumor characteristics. The purpose was.

**Fig.1:**
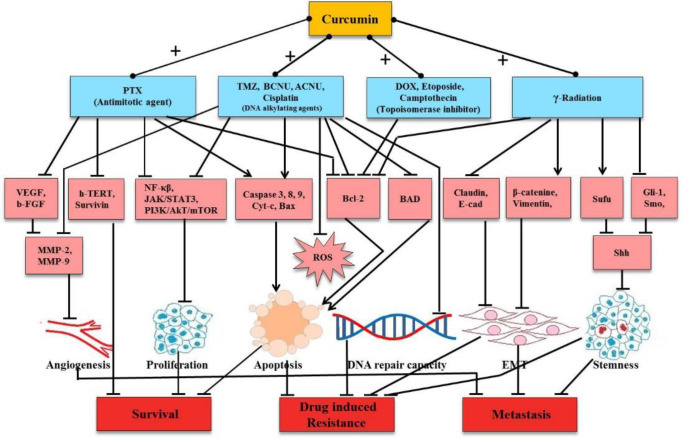
**Schematic representation of Curcumin the pleiotropic molecule, as a sensitizer and adjunct to conventional treatment modalities. **Level 1 depicts conventional therapies categorized on the basis of their individual action. Level 2 indicates the molecules and pathways targeted in the presence of CUR as the sensitizer. Level 3 specifies the cellular endpoints, and level 4 presents the expected clinical outcomes. ACNU : nimustine; BCNU: carmustine; Bax: Bcl-2-associated X protein; BAD: Bcl-2-associated death promoter; Bcl-2: B-cell lymphoma 2; bFGF: basic fibroblast growth factor; Cyt c: cytochrome-c; Dox: doxorubicin; DNA: deoxyribonucleic acid; EMT: epithelial to mesenchymal transition; GLI1: glioma-associated oncogene homolog 1; hTERT: human telomerase reverse transcriptase; JAK/STAT3: Janus kinases/signal transducer and activator of transcription 3; MMP: matrix metalloproteinases, mTOR: mammalian target of rapamycin; NFκB: nuclear factor kappa-light-chain-enhancer of activated B-cells; PI3K/Akt: phosphatidylinositol 3-kinases/ protein kinase B, PTX: paclitaxel; TMZ: temozolamide; ROS: reactive oxygen species; Sufu: suppressor of fused homolog; Shh: sonic hedgehog; Smo: smoothened homolog precursor; VEGF: vascular endothelial growth factor. ↓ (induction); ┴ (inhibition)

also to identify the gaps in research and highlight the advantages of ongoing experimental innovative interventions. This will lay the blueprint for further successful animal and human studies to bring in CUR as a licensed adjuvant in the first line of treatment for CNS tumors

**Curcumin can tackle the drug resistance mecha-nisms**

The difficulty in treating brain malignancies lies in the integral complexity of the tumor entity and also in the multiple mechanisms of drug resistance involved, namely inflammation, drug efflux, DNA damage repair activation, EMT, CSCs, etc (18,19). The approach to overcoming these barriers is to sensitize tumor cells by using CUR to potentiate the activity of onco-therapeutics via modulation of one or more mechanisms of resistance.

***Curcumin inhibits inflammation***

Inflammation is a natural immune reaction to an infection or tissue injury. However, if the cause of the initial inflammatory reaction is not addressed, acute inflammation can transit to chronic inflammation (20). Chronic inflammation can further trigger oncogenesis in the brain by affecting the behavior of neural stem cells (NSCs) and oligodendrocyte precursors (OPCs). Mediators produced during inflammation can influence NSC migration and uncontrolled proliferation of both (21, 22). Several experimental settings have established CUR as an anti-inflammatory, it pedals inflammation by suppressing the transcription of nuclear factor kappa B (*NF-κB*) (23) and other genes involved in this signaling cascade, including cyclooxygenase (*COX-2*), adhesion molecules, interleukins (*IL-1*, *IL-6*), *TNF-α*, etc. (24,25).

***Curcumin inhibits drug efflux***

Orally administered drugs usually lack therapeutic output due to poor bioavailability and tumor-specific accumulation which is mostly attributed to the efflux of drugs by the multidrug-resistance-linked ATP binding cassette (ABC) drug transporters, leading to resistance. Compelling evidence exists, that CUR can inhibit multidrug resistance (MDR) by inhibiting the activity of ABC drug transporter family members and improving the accumulation and sensitivity of tumor cells to chemotherapy (26, 27).

***Curcumin inhibits pathways involved in DNA damage repair in cancer cells***

Treatment of brain malignancies with alkylating chemotherapeutics like temozolomide (TMZ), carmustine (BCNU), and nimustine (ACNU) results in cellular resistance following the activation of fanconi anemia (FA) pathway and/or methylguanine methyltransferase (MGMT) (28, 29). FA is a DNA repair pathway (30) while MGMT, a DNA repair enzyme that repairs cytotoxic lesions created by alkylating agents (29). FA complementation group D2 (FANCD2) protein of the FA DNA repair pathway is upregulated on exposure to alkylating agents in GB. At a clinically achievable concentration of 5 μM, CUR was demonstrated to inhibit this protein in GB cells (29, 31). CUR has also been shown to trigger DNA damage and inhibit the expression of MGMT (32).

***Curcumin inhibits CSC’s and EMT phenomenon***

Resistance to standard treatment modalities is also attributed to the existence of two other factors namely CSCs or cancer stem-like cells (CSLCs) and EMT. CSCs are self-renewing, differentiating subpopulation of cells within a tumor while EMT is a conserved but reversible phenomenon of malignancy. EMT promotes stemness by changing the tumor microenvironment, resulting in an increasing number of circulating tumor cells (CTCs) that undergo transformation leading to the development of CSCs (33), thereby contributing to relapse and metastasis. CUR at sub-lethal doses has been demonstrated to inhibit self-renewing characteristics of glial stem cells (34). It has also been reported to prevent EMT in radio-resistant glial cells (35).

**CUR in combination with other treatment**

**modlities for regressing brain tumors**

***CUR as a sensitizer for drugs that can cross the BBB***

CUR as an agent for the treatment of brain cancers has shown promising results in preclinical studies and there is accumulating evidence that the compound serves to potentiate standard chemotherapeutic agents both in *in vitro* and *in vivo* models (Table 1). TMZ is used in the clinic as the first-line chemotherapeutic option for glioma and other brain malignancies like pituitary tumors, medulloblastomas, and brain metastases (36, 37). TMZ can cross the BBB, but its therapeutic efficacy becomes limited due to the chemo-resistance and toxicity elicited at the clinically used dosage. Studies on the treatment in glioma cell lines and primary glioma cultures have reported that CUR inhibits the FA DNA repair pathway thereby overcoming chemoresistance in brain tumors treated with alkylating agents such as TMZ, as discussed above (29). Also, simultaneous CUR administration has been shown to amplify the anti-proliferative and apoptotic potential of TMZ in glioma models. CUR was demonstrated to augment the therapeutic response of TMZ in GB cells and xenograft mouse model by concurrently generating reactive oxygen species (ROS) levels and enhancing apoptosis via blocking protein kinase B/Mammalian target of rapamycin (AKT/mTOR) signaling, decreasing the expression of Bcl-2-associated death (BAD) promoter (38) and also via up-regulation of miR146a and inactivation of Nuclear factor kappa-light-chain-enhancer of activated B cells (NFκB) signaling (39). CUR was also shown to significantly reduce the over-expression of connexin (*Cx43*), a gap junction assembly protein, by about 40% and increase TMZ-induced apoptosis from 4% to 8% in TMZ-resistant GB cells via the ubiquitin-proteasome pathway (4) thus potentiating the action of TMZ. CUR was also reported as a beneficial adjuvant with TMZ and etoposide as this triple combination induced apoptosis by downregulatiing *p10* and *p53* mRNAs with a concomitant rise in Bcl-2-associated X protein/B-cell lymphoma 2 (*BAX*/*Bcl-2*) mRNA ratio at IC_50_ doses, in comparison with the individual or the dual drug combination in GB and medulloblastoma cell lines (40).

Similarly, CUR was reported to work as an adjunct to another alkylating agent ACNU. The co-treatment led to the suppression of GB cell proliferation, clone-forming capacity, invasion, migration, induction of apoptosis, and cell-cycle arrest in the G2/M phase. This synergistic effect was mediated through concurrent activation of the cytochrome C/caspase-dependent apoptotic pathway, inactivation of phosphatidylinositol 3-kinases/ protein kinase B (PI3K/AKT), and NF-κB/COX-2 signaling cascades (41).

**Table 1:** Studies presenting mechanism of CUR action in combination with radio/ chemotherapeutics at effective dosages in brain cancer cells/ xenograft models

**CUR**	**Chemo/Radio therapy**	**Assay system**	**Parameters ** **assessed **	**Endpoint** **measured **	**Reference**
5 μM	TMZ (50 μM)	CRL2020, U87, CRL2366 A172, T98G, and HTB16 (FA-deficient)	↓FANCD2 nuclear foci ↓FANCD2 mono-ubiquitination	↓FA DNA repair pathway ↓resistance induced by alkylating agents	[29]
5 μM	BCNU (50 μM)	CRL2020, U87, CRL2366 A172, T98G, and HTB16 (FA-deficient)	↓FANCD2 nuclear foci ↓FANCD2 mono-ubiquitination	↓FA DNA repair pathway ↓resistance induced by alkylating agents	[29]
5 μM	TMZ (50μM)	FA-proficient (U87)/deficient (U138) cells, primary GBM cell cultures	↓FANCD2 nuclear foci ↓FANCD2 mono-ubiquitination	↓FA DNA repair pathway ↓resistance induced by alkylating agents	[31]
5 μM	BCNU (50 μM)	FA-proficient (U87)/deficient (U138) cells, primary GBM cell cultures	↓FANCD2 nuclear foci ↓FANCD2 mono-ubiquitination	↓FA DNA repair pathway ↓resistance induced by alkylating agents	[31]
1.25 µg/ml	TMZ (15.63 µg/ml)	U87MG	↑ROS, ↓AKT/mTOR, ↓phosphorylation of BAD	↓survival ↑apoptosis	[38]
1.25 µg/ml	TMZ (15.63 µg/ml)	U87MG xenograft mouse model	↓AKT/mTOR, ↓phosphorylation of BAD	↑apoptosis ↓tumor volume, ↓tumor weight	[38]
20 µM	TMZ (100 µM)	U87MG	↑miR146a, ↓NFκB	↓cell proliferation ↑apoptosis	[39]
10 µM	TMZ (100, 250 μM)	U251, U87	↓Cx43	↓viability ↑apoptosis	[4]
20 μM	ACNU (100μM)	U118MG, U87MG, U251MG	↑Cyt c, ↑cleaved caspase 3,↑BAX/BCL-2, ↓p-PI3K/Akt, ↓NF-κB/COX-2, ↓CDK1, ↓cyclin A, ↓cyclin B, ↑G2/M phase arrest of cells, ↓MMP2/9, ↓N-cadherin, ↓Vimentin	↑apoptosis ↓viability ↓clone forming capacity ↓invasion ↓migration	[41]
5 μ	Bromocriptine (0.01 μ	rat lactotroph pituitary cell lines (GH3 and MMQ)	↓proliferation	↑growth inhibition	[42]
25 μmol/L	Dox	T98G, U87MG cells	↑cytotoxicity, ↑DNA fragmentation	↓cell proliferation ↑apoptosis	[43]
25 μmol/L	Camptothecin	T98G, U87MG cells	↑cytotoxicity, ↑DNA fragmentation	↓cell proliferation ↑apoptosis	[43]
25 μmol/L	Etoposide	T98G, U87MG cells	↑cytotoxicity, ↑DNA fragmentation	↓cell proliferation ↑apoptosis	[43]
25 μmol/L	Cisplatin (50 μmol/L)	T98G, U87MG cells	↑cytotoxicity, ↑DNA fragmentation ↑DNA damage ↓clonogenecity	↓cell proliferation ↑apoptosis ↓DNA repair capacity	[43]
25 μmol/L	γ-irradiation (5 Gy)	T98G, U87MG cells	↑cell death	↓viability	[43]
15 and 20 µM	γ-irradiation (1-12Gy)	F98 GB cells	↑G2/M	↓viability ↓clone forming capacity	[52]
120 mg/2 mL/kg	γ-irradiation (6 Gy)	triple-reporter F98/FGT glioma-bearing rat model		↓tumor growth ↑survival	[52]
20 µM	γ-irradiation (18Gy)	LN229 and U251	↓Gli1,↑Sufu, ↓cell migration, ↓invasion, ↓E-cadherin, ↑vimentin, ↑β-catenin, ↓claudin	↓cell proliferation, ↑early apoptosis, ↓EMT, ↓Hedgehog signaling pathway	[35]
60 mg/kg	γ-irradiation (18Gy)	nude mice with glioma	↓E-cadherin, ↑vimentin	↓tumor growth, ↓EMT	[35]

In the case of prolactinoma (a functional pituitary adenoma), the first line of therapy is bromocriptine, a dopamine D2 receptor agonist; yet intolerance and resistance to this drug are inexorable. In this case, CUR was seen to augment the growth-inhibitory effect of bromocriptine in rat lactotroph tumor cell lines, suggesting its clinical usefulness in the management of prolactinomas (42).

***CUR as a sensitizer for*** ***other repurposed chemo-therapeutic agents***

Apart from the above-mentioned drugs, some other therapeutics have been tested in conjunction with CUR *in vitro* to assess their anti-proliferative and apoptosis-inducing efficacy in brain malignancies (Table 1). A study reported that CUR potentiates the action of cisplatin in brain tumors. It was experimentally shown to synergize with cisplatin to enhance the comet positive cells (signifying DNA damage), apoptosis and produced a more pronounced cellular injury in glioma cells (43). The combination triggered cytotoxic death via apoptosis by downregulating *BCL-2* in medullo-blastoma cells (44). When other chemo-drugs such as doxorubicin (dox), etoposide, and camptothecin were individually paired with CUR, they produced increased cytotoxicity and apoptosis in glioma cell lines (13, 43). Researchers have also explored the anti-neoplastic ability of CUR and paclitaxel against highly chemo-resistant human GB cells, glioma stem cells (GSCs), and C6 mouse glioma cells. They found that the combination induced apoptosis and down-regulated the expression of molecules involved in promoting cell survival, invasion, proliferation, and angiogenesis (45, 46). Castonguay and colleagues investigated the combination of their most potent ruthenium-letrozole complex (individual entities possess anti-cancerous properties) with CUR and noted that complex 5•Let and CUR synergistically induce autophagic cell death in GB cells (47).

***CUR as a sensitizer for radiotherapy***

In addition to the synergistic effect of CUR with chemotherapy, the polyphenol has also exhibited plausible utility in sensitizing glioma cells to radiation-induced cell death (Table 1). When radio-resistant human glioma cells were pre-treated with CUR followed by irradiation, it resulted in a significantly enhanced cell death (43). In another set of experiments on medulloblastoma cells, CUR boosted the efficacy of γ- irradiation by triggering apoptosis via downregulation of anti-apoptotic protein, BCL-2 (44). CUR was also reported to trigger G2/M cell cycle arrest and sensitize the F98 GB cells to radiation *in vitro*. It synergistically enhanced the effects of radiotherapy by suppressing the growth of both transduced glioma cells and the brain tumor. CUR also prolonged the overall survival in orthotopic F-98/FGT (firefly luciferase; enhanced green fluorescent protein; thymidine kinase) glioma-bearing rat model, establishing it as a potent radiosensitizer (48). CUR was reported by Meng and co-workers to augment the effect of γ-irradiation by suppressing cell proliferation, migration, invasion, and inducing apoptosis in both *in vitro* and *in vivo* glioma models (35). The group also proposed that CUR could rescue the EMT process induced by γ-irradiation via inhibition of the hedgehog signaling pathway and modulation of EMT markers, a finding which has key clinical implications. All the above-mentioned studies establish the role of CUR as a cogent sensitizer of available treatment modalities.

***CUR in combination with small-molecule onco-therapeutics***

Apart from the existing chemo-drugs, few other molecules with anti-tumor efficacy have also been tried and tested in combination with CUR (Table 2). CUR sensitized glioma cells to tumor necrosis factor-related apoptosis-inducing ligand (TRAIL/ Apo2) resulting in increased apoptosis by arresting hypo-diploid glioma cells in the sub G1 phase, initiating the release of mitochondrial cytochrome -C and cleavage of procaspases-3, 8, 9 (49). The co-treatment of CUR with tyrphostin (small-molecular-weight tyrosine kinase inhibitors) significantly decreased ROS, increased the cytotoxic effect, and stimulated apoptosis in GB cells (50). 

***CUR along with gene therapy***

Another alternate treatment approach cited in the literature is the combination of tumor suppressor miRNA with CUR (Table 2). It is known that cancer cells have signature miRNA expression profiles. miRNA replacement therapy can restore a loss-of-function in cancer cells by the reintroduction of a tumor suppressor miRNA, to restore the deranged cellular cascades (51). Glioma cells transfected with miR-326 mimics (tumor suppressor miRNA which is lowly expressed in the glioma) and concurrently treated with CUR, exhibited significant inhibition of proliferation and migration, with a concomitant rise in apoptosis via down-regulation of sonic hedgehog (Shh) signaling cascade. Additionally, the co-treatment reduced tumor volume and prolonged survival in the glioma xenograft mouse model as compared to the individual molecules (52). Similarly, miR-378 is documented to be down-regulated in GB (53). Li and coworkers reported that transfection of GB cells with miR-378 mimics led to its overexpression, significant inhibition of cell proliferation and induction of apoptosis via increased phosphorylation of p38 and upregulation of Bax. *In vivo* extension of this group’s work indicated that miR-378 and CUR in co-therapy were more effective in inhibiting tumor growth in a mouse xenograft model (54).

**Increasing the bioavailability of CUR using nanoparticles**

The most intimidating task for a chemotherapeutic used in the treatment of brain malignancies is crossing the BBB to facilitate effective drug dose accumulation in the brain. Despite its promising pharmaceutical characteristics CUR has some major drawbacks limiting its application as an anti-cancer therapeutic agent. CUR is known to possess the ability to cross the BBB, evident from an *in vitro* experiment on the brain- capillary model (55). Its poor physicochemical properties namely low water solubility and enzymatic conversion to glucuronide derivatives, and rapid clearance from circulation result in poor bioavailability, thereby limiting its clinical utility (56). Over the years, several strategies have been applied to overcome these hitches; to enhance the solubility of CUR and prevent its enzymatic degradation (57, 58). The strategy in trend to address this issue is the use of tailored nanoparticles to competently ferry CUR along with other drugs or bioactive molecules across the BBB, to localize in the tumor at the effective therapeutic concentration. This technique efficaciously localizes drugs *in situ* and helps overcome MDR. CUR has been formulated with many nano-carriers such as polymeric nanoparticles, liposomes, nanoemulsions, solid lipid nanoparticles, and micelles (59).

**Table 2:** Measured outcomes of CUR in combination with other molecules

**CUR **	**molecules**	**Assay system**	**Parameters assessed **	**Outcome ** **measured **	**Reference**
5-20 μM	TRAIL (5ng/mL)	U251MG and U87MG glioma cells	↑hypo-diploid cells in sub G1 cell cycle phase, ↑cleavage of procaspases-3, 8, 9, ↑release of cyt-c from mitochondria	↓viability ↑apoptosis	[49]
5–12 mM	Tyrphostins (1–20 mM AG494) and (0.1–8mM AG1478)	LN229 glioma cells	↑cytotoxicity, ↑caspase 3 and 7, ↑cell cycle arrest in G1/G0 phase, ↑comet positive cells	↓viability ↑apoptosis ↓ROS ↑DNA damage ↓DNA repair	[50]
10 -40 μM	miR-326	U251 and U87	↑caspase-3, ↑cleaved PARP1, ↑caspase-8, ↓MCL1, ↓bcl-xl, ↓RIP1 ↓SHH/GLI1 ↓Nestin ↓CD133	↓proliferation ↓clone forming capacity ↑apoptosis	[52]
60 mg/kg	miR-326	BALB/c-nude mice		↓tumor volume, ↑survival	[52]
5-50 μM	miR-378	U87-miR-378	↑phosphorylation of p-38, ↑Bax, ↓PCNA	↓viability ↑apoptosis ↓clone forming capacity	[54]
60 mg/kg and 120 mg/kg	miR-378	SCID mice	↑phosphorylation of p-38	↓tumor growth	[54]

cleaved PARP1 (cleaved anti-poly ADP ribose polymerase 1), DNA (Deoxyribonucleic acid), ROS (reactive oxygen species), MCL1 (myeloid cell leukemia 1), RIP-1(receptor-interacting serine/threonine-protein kinase 1), miR (MicroRNAs), PCNA (proliferating cell nuclear antigen), TRAIL (tumor necrosis factor -related apoptosis-inducing ligand). ↑ (high expression), ↓ (low expression)

***Nanoparticle mediated delivery of CUR and other drugs***

The use of drugs loaded onto nanoparticles is enticing the attention of the neuro-oncology fraternity, due to its exceptional potential for targeted brain delivery (Table 3). The combination of CUR and rapamycin-loaded micelles exhibited ∼3.3 times higher uptake than the individual drugs or a mixture of the native drugs when assayed in T98 GB cells *in vitro*. This combination also down-regulated P13/AKT and serine/threonine kinase proteins, enhanced mitochondrial membrane potential depolarization which increased apoptosis (60). Similarly, the combined delivery of CUR and the herpes simplex virus thymidine kinase (*HSVtk*) gene using peptide micelle R7L10 as a carrier, efficiently induced apoptosis in C6 GB cells *in vitro* and in the xenograft mouse model. The combination also reduced tumor size without significant systemic toxicity in the animal GB model (61).

However, a pharmacokinetic study conducted in 2016 sparked greater interest in the use of nanoformulations of CUR. The investigators enrolled 10 GB human subjects and orally administered a micellar-curcuminoid preparation which achieved measurable concentrations of total curcuminoids in the tumor tissue and serum, validating its therapeutic utility (62). Transfection of U-87MG GB cells with *p53*-containing vector (for *p53* overexpression) and concurrent treatment with dendrosomal CUR (a nano-formulation) resulted in an increased expression of growth arrest and DNA damage-45 (*GADD45*). It also simultaneously reduced the expression of *NF-κB* and *c-Myc*, leading to decreased cell viability and increased apoptosis (63). Microemulsions (ME, nano-sized dispersion) of CUR with docosahexaenoic acid (DHA) -rich oil, were seen to dramatically lower IC_50_ values in GB cell lines. The formulation was administered intravenously and intra-nasally for targeted delivery to the rat brain. Though both the intravenous and intranasal delivery routes were effective, concentrations in the brain following intranasal administration was substa-ntially higher and sustained. The higher brain accumulation of CUR-DHA MEs was ascribed to a greater targeting efficacy augmented by DHA-mediated transport across the BBB. Serum biochemistry, hematology, histopathology, and nasal toxicity records confirmed the safety profile of the ME formulation (64). When nanomicellar-CUR in combination with TMZ was tried out, it significantly decreased the invasion and migration of U87 cells. The combination considerably increased the expression of autophagy-related proteins (LC3-I and LC3-II; microtubule-associated protein 1A/1B-light chain 1 and 2) and apoptosis-related proteins (Bcl-2 and caspase 8) while Bax expression decreased substantially. The combination also reduced the expression of β-catenin, cyclin D1, Twist, and ZEB1 (Wnt pathway-associated genes) (65). Recently, a ME-based TMZ and CUR co-loaded nanostructured lipid carrier was shown to possess enhanced synergistic inhibitory effects on glioma cells compared to single drug-loaded nano-carriers. The combination induced S phase cell cycle arrest and triggered apoptosis. Effective accumulation was observed in the brain and at tumor sites, with no toxicity to normal cells and vital organs *in vivo* when used at the therapeutic dosage (66). Tan and workers prepared a dual combination of CUR and miR21ASO (miR21 antisense-oligonucleotide) loaded onto deoxycholic acid-conjugated polyethy-leneimine micelles. Its administration reduced miR21 levels and induced apoptosis in GB cells. In intracranial rat tumor models, the complex regressed tumor volume and induced the expression of tumor suppressor genes phosphatase and tensin homolog (*PTEN*) and programmed cell death protein 4 (*PDCD4*); which were earlier inhibited in miR21 expressing GB cells (67).

**Nanoparticle mediated delivery of CUR and other natural products**

Apart from the nanoformulation-based studies above, CUR has also been delivered with some natural plant compounds (Table 3). Liposome encapsulated polyphenol trio-tricurin was developed using CUR and two other polyphenols namely, resveratrol and epigallocatechin gallate. The experiment demonstrated that the liposomal delivery system of trio-polyphenols induced apoptosis in the GB cells and GSCs via p53 activation. Tricurin also stimulated intrinsic immunity, by repolarizing tumor-associated microglia/macrophages to tumoricidal state and eliciting an intra-tumor deployment of activated natural killer cells in the GB mouse model (68). Maiti and colleagues showed that co-treatment of solid-lipid CUR and berberine considerably elevated DNA fragmentation, apoptotic death, mitochondrial degeneration, and significantly decreased ATP levels in GB cell lines in comparison to their single treatments. The co-treatment also inhibited the PI3K/Akt/mTOR pathway more proficiently compared to both the agents individually (69).

**Effective brain tumor targeting**

***Curcumin tagged to antibodies***

It is postulated that antibody-mediated homing of cancer cells would result in higher levels of a drug at the tumor site, thus intensifying CUR’s effectiveness (Table 4). Langone and co-workers while working on melanoma-induced brain metastasis linked CUR to the melanoma-specific Muc18 antibody (cell surface protein MUC18 antibody) to increase its targeting effectiveness. The CUR-antibody adduct was exceedingly competent in killing melanoma cells in culture via inhibition of signaling proteins, NF-kB, and Akt. Furthermore, they created a lab model for brain metastasis by implanting murine melanoma B16F10 cells in the right frontal lobe of the syngeneic mouse brain. Intracranially infused CUR-antibody adduct dramatically decreased tumor size and increased survival in mice, thereby decimating melanoma-induced metastatic brain tumors (70). In another attempt to augment delivery, the group covalently attached CUR to the GB-specific CD68 antibody which substantially elevated its anti-proliferative efficacy as seen *in vitro* glioma cells. Intracranial CUR-CD68 adduct infusions followed by peripheral injections of CUR in brain tumor-bearing mice led to a decline in tumor load and extended their longevity. The hematoxylin-stained brain sections showed scar tissue with no apparent inflammation in the treated mice, indicating adduct-mediated anti-tumor efficacy (71).

Daniel and the group reviewed prior studies to establish that transferrin receptors (TfR) are expressed abundantly in human cancer tissues and the anti-TfR antibody can inhibit cell growth in highly proliferating malignant cells by blocking its receptors and interfering with the iron uptake (72). In the light of this understanding, Wen and his team looked into the combined effect of anti-TfR antibody (7579 mAb) and CUR in glioma cell lines. A synergistic effect was reported concerning tumor growth suppression and the induction of cellular necrosis (73). Akin to their work, Sarisozen and co-workers developed an all-in-one nano-formulation for co-delivery of Dox and CUR incorporated into poly (ethylene glycol) – phosphatidylethanolamine conjugate based polymeric micelles. It was then tagged with glucose transporter type 1 (GLUT1) antibody scFv (single chain fragment variable) to facilitate BBB entry and specifically target GB cells. The formulation improved the synergistic action in terms of deeper penetration into the 3D spheroids, increased nuclear localization of Dox and its DNA intercalation, increased caspase 3/7 activation, which resulted in successful apoptosis, and increased tumor cytotoxicity (74).

**Table 3:** Preclinical studies on mechanism of CUR action in combination with therapeutics assisted by nano-carriers

**CUR **	**Other drug/moleule**	**Nano formulation**	**Assay system**	**Parameters ** **assessed **	**Outcome ** **measured **	**Reference**
CUR : Rapamycin 1 : 1		MePEG/PCLmicelle	T98G U373	↓NFκB, ↑mTOR ↓Akt ↓COX-2, ↓survivi ↑cleaved caspase -3	↓viability ↑apoptosis	[60]
CUR: : HSVTK 5 : 1		R7L10 peptide micelles	C6 rat GB and HEK293	↑tunel positive cells	↓viability ↑apoptosis	[61]
CUR: : HSVTK 5 : 1		R7L10 peptide micelles	Balb/c nude mice		↓tumor volume	[61]
27.5 μM	p-53 containing vector (100 ng/μl)	DM	U87-MG glioma cells	↑GADD45, ↓NF-κB, ↓c-myc	↓viability ↑apoptosis	[63]
5 mg/mL	DHA rich oil	ME (1mg/ml)	U87MG	↑cytotoxicity	↓viability	[64]
5 mg/mL	DHA rich oil	ME (1mg/ml)	Sprague-Dawley (SD) rats	↑plasma levels, ↑transport across the BBB	↑brain concentration	[64]
50 μM	TMZ 20,50μM	Micellar CUR	U87	↑Beclin 1, ↑LC3-I,↑ LC3-II,↑Bcl-2 ↑caspase 8, ↓BAX, ↓β-catenin, ↓cyclin D1, ↓Twist, ↓ZEB1	↓viability ↑autophagy ↑apoptosis ↓Wnt sinaling ↓invasion ↓migration	[65]
CUR :TMZ 2 :1		ME	Rat C6 glioma and human brain glial normal cells C6 GB cells BALB nude mice	↑S phase cell cycle arrest associated with induced apoptosis. ↑drug accumulation at tumor site	↓viability of C6 cells ↑viability of normal cells ↑apoptosis ↓ side effects ↓tumor volume	[66]
CUR :miR21ASO 9 :1		DP-Cur micelle 1:9 10 μl	C6 GB cells	↓miR21	↑intracellular delivery ↑apoptosis	[67]
CUR :miR21ASO 9 :1		DP-Cur micelle 1:9 10 μl	C6 GB cells implanted Sprague Dawley rat	↑PDCD4 ↑PTEN	↓tumor volume	
1.28 mM	Resveratrol (4 mM) + Epicatechin-gallate (320 μM)	Liposome (10μM	GL261 mouse cells	↑p53, ↑caspase3 ↓CD133, ↓SOX-2	↓viability ↑clone forming capacity ↑apoptosis ↓stemness	[68]
1.28 mM	Resveratrol (4 mM) + Epicatechin-gallate (320 μM)	Liposome (200 µL)	GL261-implanted GBM mouse model	↑conversion of M2 associated microglia/macrophages into M1-like phenotype ↓CD68 ↓CD133 ↓NK cells	↓tumor cell ↑apoptosis ↓stemness ↑survival	[68]
20 μM	Berberine 100 μM	solid lipid CUR particles	U-87MG, U-251MG SHSY5Y (human tissue derived NB cell line)	↑DNA fragmentation, ↑Comet positive cells, ↓ATP, ↓ MMP, ↓PI3K/Akt/ mTOR, ↑Bax, ↑cytochrome-c, ↑cleaved caspase-3, ↓Bcl-2	↓proliferation ↑ROS ↑apoptosis	[69]

BBB (blood brain barrier), DHA (Docosahexaenoic acid), DM (Dendrosomal micelle), DP (deoxycholic acid-conjugated polyethylenimine). Dox (Doxorubicin), GADD45 (The Growth Arrest and DNA Damage inducible -45), HEK293 (human embryonic kidney 293), HSVtk (herpes simplex virus thymidine kinase), MePEG/PCL (Methoxy poly(ethylene glycol)-ε-poly(caprolactone) diblock copolymers),ME (Microemulsion), miR21ASO (antisense-oligonucleotide against miR-21), MMP (mitochondrial membrane potential); MNP (Magnetic nanoparticle, NFκB (nuclear factor kappa-light-chain-enhancer of activated B cells), PDCD4 (programmed cell death 4). PI3K/Akt phosphatidylinositol 3-kinases, PTEN (phosphatase and tensin homologue),TMZ (temozolamide), U87-Luc (U87 glioma cells genetically marked with a firefly luciferase reporter gene). ↑ (high expression), ↓ (low expression).

***CUR delivered with other drugs under magnetic influence***

Various research groups in the past decade have experimented with different brain targeting strategies for co-delivery of CUR and standard chemo-drug using nanoparticles under the magnetic influence with or without ligands; to achieve better treatment endpoints (Table 4). A formulation of CUR and TMZ loaded magnetic nanoparticles (MNPs) attained a higher synergistic cytotoxic effect than single drug-loaded MNPs or native drugs when assessed in monolayer cultures and tumor spheroid models of GB. The co-treatment ensured profound penetration of the MNPs into the spheroids (75). 

Fang and co-workers fabricated lactoferrin ligand (Lf)-tethered magnetic double emulsion nanocapsules from polyvinyl alcohol, polyacrylic acid, and iron oxide nanoparticles to simultaneously load Dox (hydrophilic) and CUR (hydrophobic). The preparation was complemented with an external magnetic field (MF) as an additional tool for targeting. This dual-targeting led to an elevated CUR-Dox concentration in the cancer cells *in vitro*, resulting in higher cytotoxicity. An intravenous injection of this formulation given to heterotrophic brain tumor-bearing mice under MF influence amplified the targeting potency of Dox and CUR at the tumor site and suppressed its growth more proficiently than the individual agents. The guided MF served to rapidly internalize nanoparticles with proficient accumulation in both the preclinical models (76). 

Similarly, working on the dual-targeting strategy, Cui and colleagues formulated a transferrin receptor-binding peptide T7-modified magnetic poly (lactic-co-glycolic acid) nanoparticle system which had paclitaxel and CUR loaded together. Combined drug-laden nano-cargos were highly effective in inhibiting proliferation, clonogenic survival, and inducing apoptosis and G2/M cell cycle arrest in glioma cells. This ligand-mediated and magnetically guided dual targeting method improved transport across the BBB as seen in both *in vitro* models of the BBB and orthotopic glioma-bearing mice. The adjunct therapy led to a ten times higher cellular uptake in the GB cells compared to non-targeted nanoparticles. It also improved survival in the mice model (77). 

## Clinical report

There are various clinical trials of CUR in different cancers (78). Despite substantial pre-clinical data available on brain tumors, clinical reports/trials documenting CUR as a therapeutic singularly or as an adjuvant are scarce (56, 79). As of date, only a single clinical case report has documented the use of CUR as an adjuvant in brain malignancy. This case presentation is of a 60-year- old woman with recurrent GB. The patient had undergone a first intervention as surgery along with radiotherapy and adjuvant TMZ on her diagnosis. On first recurrence, she again had surgery and chemotherapy with fotemustine. However, when her tumor recurred a second time and she was deemed eligible only for palliative care, she was administered 100 mg CUR four times a day, together with the anti-angiogenic drug bevacizumab, an anti-edemic herb *Boswellia serrate*, supported by capacitive hyperthermia. This third line therapy regressed her tumor size to almost half, achieving improvement in the quality of life of the patient and also extended her survival by 6 months (80). This study suggests that CUR should be taken up for clinical trial along with TMZ as first line therapy which will tackle chemoresistance and give a better outcome in terms of progression-free survival.

**Table 4:** Cellular outcome of CUR coupled with and without antibodies/ligands under magnetic targeting

**CUR **	**Other drug**	**Antibody/** **ligand**	**Assay system**	**Parameters assessed **	**Outcome ** **measured **	**Reference**
90 nM (IC_50_)	-	Muc18Ab (20μl)	B16F10 melanoma cells	↓NF-kB, ↓P-Akt-1, ↓caspase-3/7 activation	↓proliferation	[70]
6.7-20.1 pmol (final concentration in the tumor 167–335 nM)	-	Muc18Ab (20μl)	syngeneic C57BL6 mice implanted with B16F10 cells	↓tumor load ↓tumor size	↑survival	[70]
50 µM	-	GB-specific CD68 antibody (20 µg)	GL261 mouse glioma cells, T98G and U87MG glioma cells	↓NF-κB, ↓P-Akt1, ↓VEGF, ↓cyclin D1, ↓BClxL	↑cell death ↑angiogenesis	[71]
667 µM	-	GB-specific CD68 antibody	GL261-implanted adult C57BL/6 male mice	↓tumor load	↑survival	[71]
40 μg/mL	-	Anti-TfR mAb 7579 (100μg/ mL)	A172 and U87-MG cells	↑cytotoxicity, ↑S-phase arrest, ↑G2/M arrest	↑necrosis ↓proliferation	[73]
1.1 mg/ml (7.1% w/w)	Dox (0.081 mg/ml, 0.6% w/w)	PM with GLUT1 antibody (1 mg/ml)	U87MG glioma cells	↑caspase 3/7, ↑nuclear colocalization of DOX	↓viability ↑apoptosis	[74]
1.1 mg/ml (7.1% w/w)	Dox (0.081 mg/ml, 0.6% w/w)	PM with GLUT1 antibody (1 mg/ml)	3D spheroid model		↑penetration ↑treatment efficacy	[74]
5 mg	TMZ (5mg)	MNP	2-D monolayer 3-D tumor spheroid culture of T-98G glioma cells	↑penetration, ↑cellular uptake, ↑cytotoxicity, ↑nuclear fragmentation	↓proliferation ↑enhanced accumulation, ↑cell death	[75]
5 μg/ mL	Dox (5μg /mL)	Lactoferrin ligands and MNP’s	RG2 rat glioma cells	↑uptake, ↑accumulation ↓viability	↑cell death	[76]
20.8 μg/ kg	Dox (15.6 μg/ kg)	Lactoferrin ligands and MNP’s	BALB/c female nude mice implanted with RG2 cells	↑drug accumulation	↓tumor volume ↑survival	[76]
30 nM	Paclitaxel (10nM)	T7-modified PLGA –MNP system	U87-Luc	↑cell cycle arrest	↑apoptosis	[77]
30 nM	Paclitaxel (10nM)	T7-modified PLGA –MNP system	mice bearing orthotopic glioma	↑cellular uptake, ↑brain accumulation	↓adverse effects ↑survival rate ↓tumor growth	[77]

Anti-TfR mAb (Anti-Transferrin Receptor monoclonal Antibody), BClxL (B-cell lymphoma-extra large), Bcl-2 (B-cell lymphoma 2); MNP (Magnetic nanoparticle), MUC18 Ab (cell surface protein MUC18 antibody), NFκB (nuclear factor kappa-light-chain-enhancer of activate d B cells), PI3K/Akt phosphatidylinositol 3-kinases/ protein kinase B, PM with GLUT1 antibody (PEG- PE-based polymeric micelles with GLUT1 antibody single chain fragment variable), T7-modified PLGA-MNP system [transferrin receptor-binding peptide T7-modified poly(lactic-co-glycolic acid) magnetic nanoparticle system magnetic nanoparticles], VEGF (vascular endothelial growth factor). ↑ (high expression), ↓ (low expression).

Similarly, a few other compounds which have anti-inflammatory and antiproliferative properties along with safety data in clinical trials such as 2-deoxyglucose (81), resveratrol (82), artemisinins (83), ursolic acid (84), etc. should also be tried and tested in combination with CUR to increase the horizon of re-tracking de-regulate pathways in brain tumors to get improved treatment outcomes.

**Limitations of previous work and future prospects**

Pharmacokinetic deficiency in terms of gastrointestinal absorption and bioavailability is its most critical limitation and is a major obstacle in its use as a licensed drug (85). To address this issue further animal study should be rigorously designed for targeted delivery of CUR to the tumor site. Another point to ponder is why post innumerable preclinical studies, regulatory approvals for the use of some very promising nano-formulations have not been taken and tested in human subjects as there is only one case study to date in brain malignancy. Caution needs to be exercised while addressing bioavailability as the structural modification of CUR during nanoparticle synthesis may lessen its efficacy. Also, the toxic solvents used in its preparation may increase its bioavailability but limit its clinical usage and are not very cost-effective (86). In light of the present critical review of literature, the strategy that has stood out as the most promising combination to be taken up for pre-clinical studies is a one-pot formulation, that is, antibody coupled with nano-CUR along with radiation and/or sub-toxic doses of chemo-drugs. This approach can help accumulate a therapeutic dosage in the brain, and also prevent rapid systemic drug clearance. All of this would be achieved without perturbing the established clinical regimen. Clinical studies should be undertaken where CUR is used as an adjunct with TMZ and radiotherapy as the first line of treatment in order to improve prognosis, quality of life, and progression-free survival.

## Conclusion

CUR is a well-established non-toxic nutraceutical with anti-cancerous properties. The present in-depth analysis highlights the anti-cancer potential of CUR which can be credited to its capability of multiple targeting of various resistance mechanisms of CNS tumors; specifically, the GSCs, which are induced by the current treatment modalities. In addition, it acts in synergism with chemo-radiotherapy thus presenting as an adjuvant that can be used for enhancing the efficacy of clinical treatment. Its incorporation into a bio-friendly delivery system(s) can enhance its systemic bioavailability further, by improving BBB passage and targeted accumulation in tumor cells. Thus, in the continuing quest for more efficient and non-toxic anticancer agents for brain tumors, CUR has emerged as an encouraging option.

## Conflict of Interest

Authors have no conflict of interest, financial or any other to disclose.
